# Impact of active tuberculosis on social mobility and its gender differences: Difference in differences using nationwide tuberculosis surveillance data and national health insurance data

**DOI:** 10.1371/journal.pone.0334961

**Published:** 2025-11-12

**Authors:** Daseul Moon, Dawoon Jeong, Young Ae Kang, Gyeong In Lee, Hongjo Choi

**Affiliations:** 1 Busan Center for Infectious Disease Control and Prevention, Pusan National University Hospital, Busan, Republic of Korea; 2 Department of Preventive Medicine, Seoul National University College of Medicine, Seoul, Republic of Korea; 3 Division of Pulmonary and Critical Care Medicine, Department of Internal Medicine, Severance Hospital, Yonsei University College of Medicine, Seoul, Republic of Korea; 4 The Korean Institute of Tuberculosis, Korean National Tuberculosis Association, Cheongju, Republic of Korea; 5 Division of Health Policy and Management, Korea University College of Health Science, Seoul, Republic of Korea; George Mason University College of Health and Human Services, UNITED STATES OF AMERICA

## Abstract

Although reducing catastrophic total costs caused by TB is a major public health concern, there is a scarcity of long-term follow-up studies on social suffering due to TB as well as studies examining gender gaps. This study aims to examine the degree of long-term change in household incomes due to active TB by gender. We created data for the TB and control groups by linking the Korean National Tuberculosis Surveillance System (KNTSS) and National Health Information Database (NHID) and covariate-adjusted propensity score matching (PSM). We created longitudinal panel data from two years before TB diagnosis (t) to two years after TB diagnosis and analyzed the changes in household income deciles by gender and group using a difference in differences (DID) model. In men, there was a clear trend of declining income since time t in the TB group (DID coefficient = −0.131 95% CI = −0.132 ~ −0.129), but there was no marked change in women. Subgroup analyses on the working-age population (20–65 years) (DID coefficient = −0.053, 95% CI = −0.096 ~ −0.010) and employee population (DID coefficient = −0.072, 95% CI = −0.110 ~ −0.034) showed a trend of declining income in the female TB group. This study showed that there is a marked trend of declining income due to the diagnosis and treatment of active TB in men but not in women. This discrepancy may be attributable to the differences in gender roles in a patriarchal society and higher possibility of women moving out of the labor market after disease. There is a pressing need for comprehensive and universal implementation of health and social protection policies to alleviate the trend of social suffering caused by disease.

## Introduction

Social causation and social selection have long been topics of debate in health inequalities research [1]. In general, change in socioeconomic status (SES) due to health is described in terms of social mobility [2]. Particularly, multiple studies have been conducted on both intra- and inter-generational social mobility since 2000 [3–6]. One of the largest body of studies on social mobility is about medical impoverishment research examining poverty or income loss induced by health shock [7,8]. Recent findings from studies on medical impoverishment suggested that health shocks can impact not only the social mobility of individuals experiencing ill health but also influence the SES of their households [9–11].

Tuberculosis (TB) has been empirically proposed to be a disease caused by poverty as well as a disease to induce poverty due to income loss [12]. To break the vicious cycle between TB and poverty, global TB strategies have begun to pay attention to the economic burden experienced by TB patients. Economic burden is typically categorized into direct costs, which primarily consist of medical expenses for treatment, and indirect costs, which include transportation expenses and income loss due to illness during the treatment process. Global TB strategies from the World Health Organization have focused primarily on achieving zero catastrophic costs due to TB in alignment with the Sustainable Development Goals [13]. Most countries design their national tuberculosis programs and policies according to this WHO’s strategy, and research follows suit. Studies analyzing the economic burden of TB at the country level have examined the catastrophic expenses experienced by TB patients and their magnitude [14]. Recently, twenty countries conducted patient cost surveys for TB patients and identified that almost half of TB patients and their households faced catastrophic costs [15].

There has been growing interest in the impoverishment experienced by TB patients due to income or livelihood loss. Studies from various settings have consistently reported income decline and job loss following TB diagnosis, including research from Zimbabwe [16], Ethiopia [17], Brazil [18], India [19], and Malawi [20]. However, most studies have limitations in that they were conducted according to the WHO tuberculosis patient cost surveys handbook guidelines [21] and analyzed cross-sectional survey results rather than longitudinal data based on national surveillance systems. Additionally, the economic burden of TB may vary according to each country’s unique policy arrangements.

Empirical evidence on the economic burden experienced by TB patients in Korea—a high-income country with universal health insurance but high TB incidence—remains limited. While there is a scarcity of empirical evidence to support its precise effects on social mobility, an empirical study with a focus on tuberculosis treatment experiences in Korea reported that there are differences in household income reduction following tuberculosis treatment according to childhood SES, and the said study reported that downward social mobility has been intensified since the 1998 financial crisis [22]. As the study used cross-sectional data, however, they were only able to present limited evidence obtained by examining changes in SES before and after disease diagnosis.

The existing literature lacks not only longitudinal studies examining dynamic changes in socioeconomic position but also gender-stratified analyses of TB’s impact on household economic mobility. The gender of whom experiencing the health problem could modify the impact of health shocks on SES [23]. Work is one of the key mechanisms that explain social mobility through the acquisition of material resources. Considering that women’s more precarious position in labor market with job insecurity, fewer benefits, and even less employment opportunities, TB diagnosis might lead to more pronounced downward social mobility for women than men. Unequal distribution of caregiving responsibilities within the family might notably decrease the SES of women than men if any family member is diagnosed with TB as well. However, findings on gender-specific social mobility of family due to poor health are ambiguous. A study conducted on British reported that inter- and intra-generational downward social mobility induced by poor health were more evident for men [6]. Another British study conducted on adolescents to follow intra-generational changes in social deprivation index observed a downward mobility but did not observe marked gender gaps [4].

Evidence on TB-induced social mobility and its gender differences is sorely lacking. To close the knowledge gap, this study enhances understanding of the dynamics of social mobility over time in tuberculosis patients and the control group using longitudinal data from Korea’s unique healthcare context. Korea represents a distinctive setting as a high-income country with universal health insurance coverage but relatively high TB incidence among OECD countries. By utilizing comprehensive data from the Korean National Tuberculosis Surveillance System and National Health Information Database, we examine the dynamics of household income changes over approximately five years surrounding TB diagnosis. Furthermore, we attempt to shed light on the gender gaps in disease-induced poverty by exploring the variances in social mobility between genders. Therefore, the study aims to identify downward, or upward household income changes of TB patients compared with non-TB groups, stratified by gender.

## Method

### Data source and study population

In this study, we used data from the 2013–2018 Korean National Tuberculosis Surveillance System (KNTSS) and National Health Information Database (NHID). The KNTSS is a nationwide registry that includes mandatory reports of all TB cases diagnosed in Korea, containing information such as diagnosis date, reporting institution, and demographic details. The NHID is a comprehensive health claims database managed by the National Health Insurance Service (NHIS), which includes sociodemographic information, medical diagnoses, prescriptions, procedures, and healthcare utilization. The two sources of data were combined by a process described in a previous study [24]. For the main study population, the TB group was defined as patients diagnosed with active TB between 2015 and 2016 and have been reported to the KNTSS. The control group was extracted from the NHID. We first stratified individuals by gender and age and extracted those with similar characteristics from those of the TB group at a ratio of 1 (TB group) to 3 (control group). The exclusion criteria for the control group were history of reporting to the KNTSS during the study period or history of healthcare utilization for TB codes (A15–A19) documented in the NHID.

### Measurement

As a time-varying variable, SES was measured using the health insurance type and health insurance premiums, a proxy for income provided in the NHID data. The NHID divided health insurance beneficiaries into deciles (1–10) based on their premiums, excluding the bottom 3% of the population who are Medical Aid beneficiaries. To include Medical Aid beneficiaries, the SES variable consisted of deciles 0–10. Decile 10 is the highest income decile, and the variable was treated as a continuous variable from 0–10. For the groups diagnosed with TB in 2015 and 2016, the year of diagnosis was set as t, and household income in years t-2, t-1, t, t + 1, and t + 2 were measured. For the control group, 2015 was set as t, and as with the TB groups, their household incomes in five years were measured. Gender was categorized as male and female and was used as a stratifier. Age was divided into 10-year units with reference to ≤ 29 years, and those aged 80 years and over were grouped as one. Disability was divided into disabled (mild and severe) and not disabled. To reduce the risk of bias caused by comorbidity severity, we measured the Charlson comorbidity index (CCI) using the method outlined in a previous study [25]. In addition, we used the ICD-10 codes to additionally detect drug-resistant TB (U84, U88.0, U88.1). For insurance eligibility, community-based health insurance beneficiaries were categorized into the self-employed insured and dependents, while work-based health insurance beneficiaries were categorized into the employed insured and dependents. Medical aid beneficiaries were divided into medical aid beneficiary and medical aid beneficiary-dependent. A panel data was created with all variables from t-2 to t + 2.

### Difference in differences model using propensity matching

The Difference-in-Differences (DID) model is widely used for analyzing policy effects, and many researchers simultaneously employ propensity score matching (PSM) to establish both the policy effect group and the counterfactual group [26]. One of the major assumptions of the DID model is the parallel trend assumption, which proves challenging to satisfy in many studies. To overcome these methodological limitations, researchers employ PSM, but it is difficult to completely eliminate bias if only adjusting for the baseline (in this study, the t-2 period). As an alternative, a method has been proposed to match the distributions of outcome variables from all time points before time t in both the experimental and control groups [27]. In this study, we attempted PSM using the method proposed by Lindner and McConnell [27], which includes covariates that may influence the categorization of the independent variables and income levels at both t-2 and t-1. Therefore, we performed PSM using a model that considers group classification as an independent variable, t-2 values for age and gender (which are time-invariant variables), and both t-2 and t-1 values for CCI and income (which are time-variant variables).

The panel DID model was established using the following equation:


$${\text{Y}}_{\text{it}}=\beta_{\text{0}}+\beta_{\text{1}}{\text{T}}_{\text{t}}+\beta_{\text{2}}{\text{I}}_{\text{i}}+\beta_{\text{3}}{\text{T}}_{\text{t}}{\text{I}}_{\text{i}}+\gamma \text{X}+\text{e},$$

In this equation, Yit is the income decile of group i at t. T_t_ is a dummy variable for time differentiating before and after t; t-2 and t-1 are pre-treatment periods, and t to t + 2 are post-treatment periods. I_i_ is a dummy variable for group classification. X is covariates, including age, disability stats, and CCI. The panel DID model was gender-stratified for analysis. To examine the characteristics of the economically active population, we additionally conducted subgroup analyses for ages 20–65 years and workers (based on having work-based health insurance in t-2). Prior to the main analysis, we conducted an event study to validate the parallel trends assumption. The event study specification adjusted for the same covariates as the main DID model.

Three sensitivity analyses were conducted to determine the risk of bias from misclassification of the three major variables—time, group, and income. First, because changes in income level due to TB disease may be delayed after the TB diagnosis, we performed the panel DID analysis with the assumption that t + 1 is the time of change. Second, groups with the extreme income values (decile 0 and decile 10) may have a grave impact on class mobility, so these two groups were excluded from the analysis. Third, drug-resistant TB requires more prolonged treatment and more costly medications compared to drug-sensitive TB, which may lead to high medical cost and indirect costs. Thus, we added a sensitivity analysis excluding drug-resistant TB.

Statistical analysis was performed using the Stata/MP version 17 (StataCorp LLC, College Station, TX, USA), and differences in the distribution of groups based on the baseline characteristics were analyzed with chi-square test or independent t-test. PSM was performed using psmatch 2 with a caliper of 0.00001. Panel DID analysis was performed using model 1 with the xtdidregress command, and to obtain robust results, model 2 was also presented with 95% CI using wild bootstrapping (random number seed = 111). Parallel trend was examined using ptrend, and grangerplot was used to examine treatment effects by time point. This study was conducted according to the 2008 Declaration of Helsinki and approved by the independent Institutional Review Board of Yonsei University Health System (IRB number: 4-2019-0917).

## Results

**Fig 1:**
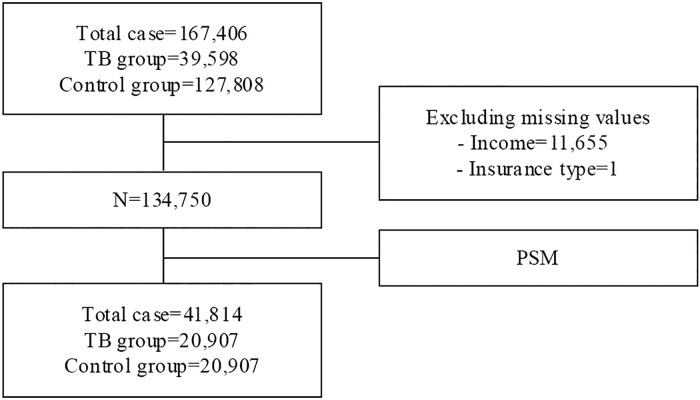
The flow of study population. TB = tuberculosis, PSM = propensity score matching.

The TB group consisted of data from 39,590 patients, and the control group consisted of data from 127,808 individuals. After PSM, a total of 41,814 participants were included in the final analysis (Fig 1). Among males, there were 12,101 in the TB group and 12,098 in the control group. Among females, there were 8,806 in the TB group and 8,809 in the control group. At the baseline, there were no significant differences in gender, age, CCI, and income level between the two groups. The distribution of other covariates was similar between groups, but the percentage of individuals with a severe disability (1.6%) and percentage of community-based health insurance members (both head of household and dependent members) were higher in the TB group compared to the control group (Table 1).

**Table 1:** Baseline (t-2 year) characteristics of the study population by gender and groups.

	Men					Women				
	Control group		TB group		p	Control group		TB group		p
	n	%	n	%		n	%	n	%	
Age										
~ 29	1,382	11.4	1,382	11.4	1.000	1,231	14.0	1,231	14.0	0.999
30-39	951	7.9	938	7.8		903	10.3	884	10.0	
40-49	1,750	14.5	1,751	14.5		997	11.3	997	11.3	
50-59	2,675	22.1	2,675	22.1		1,249	14.2	1,249	14.2	
60-69	2,203	18.2	2,203	18.2		1,010	11.5	1,007	11.4	
70-79	2,368	19.6	2,368	19.6		2,127	24.2	2,127	24.2	
80~	769	6.4	784	6.5		1,292	14.7	1,311	14.9	
Disability										
non-disabled	10,534	87.1	10,264	84.8	<0.001	7,925	90.0	7,733	87.8	<0.001
mild disabled	923	7.6	1,007	8.3		577	6.6	626	7.1	
severe disabled	641	5.3	830	6.9		307	3.5	447	5.1	
CCI										
0	6,253	51.7	6,252	51.7	1.000	4,282	48.6	4,280	48.6	1.000
1	2,108	17.4	2,110	17.4		1,841	20.9	1,842	20.9	
2	1,481	12.2	1,481	12.2		1,004	11.4	1,002	11.4	
3~	2,256	18.7	2,258	18.7		1,682	19.1	1,682	19.1	
Insurance Type										
The self-employed insured	2,802	23.2	3,179	26.3	<0.001	1,052	11.9	1,097	12.5	0.002
The self-employed insured-dependent	921	7.6	1,312	10.8		1,521	17.3	1,581	18.0	
The employed insured	3,568	29.5	2,524	20.9		1,340	15.2	1,151	13.1	
The employed insured-dependent	3,855	31.9	4,131	34.1		3,911	44.4	3,983	45.2	
Medical aid beneficiary	847	7.0	863	7.1		782	8.9	813	9.2	
Medical aid beneficiary-dependent	105	0.9	92	0.8		203	2.3	181	2.1	
	mean	SD	mean	SD	p	mean	SD	mean	SD	p
Household income	5.684	3.260	5.676	3.261	0.848	5.599	3.472	5.580	3.476	0.715

CCI = Charlson comorbidity index, SD = standard deviation.

Table 2 shows the changes in the distributions of each characteristic over time. In terms of disability, the individuals with mild disability had the highest odds for mobility; changes were observed in about 11.1% of the male control group and 12.2% of the TB group and in about 8.9% of the female control group and 12.2% of the TB group. The amount of change over time was the lowest in the group without a comorbidity, where the percentages of individuals with increased CCI were higher in the TB group compared to the control group in both male and female populations. In addition, the amount of change over time was smaller in the group with CCI ≥ 3 compared to the group with a CCI of 1 or 2. In terms of health insurance eligibility, there were marked gender gaps in the amount of change over time among the self-employed insured and the employed insured. Overall, the odds for change over time were higher in the TB group than control group. Among men, the self-employed insured showed 1.4% higher change rate in the TB group than control group, while the employed insured showed 6.8% higher change rate in the TB group than control group. Among women, the change rates for the self-employed insured and the employed insured were 4.2% and 3.5% higher in the TB group than control group.

**Table 2:** Panel structure of covariates during the study period by gender and groups.

	Men				Women			
	Control group		TB group		Control group		TB group	
	Between %	Within %	Between %	Within %	Between %	Within %	Between %	Within %
Age								
~ 29	11.42	90.47	11.42	89.68	13.97	86.31	13.98	86.59
30-39	10.16	73.35	10.07	71.58	14.03	71.65	13.75	71.8
40-49	17.49	75.66	17.83	71.15	15.54	73.04	15.34	74.24
50-59	27.57	76.6	28.89	72.66	18.45	75.84	18.23	73.51
60-69	25.86	74.43	27.46	71.62	16.23	72.79	17.11	69.43
70-79	25.54	75.81	26.25	75.63	28.31	77.2	28.75	76.4
80~	12.24	75.63	12.22	76.52	23.02	81.96	24.35	79.61
Disability								
Non-disabled	87.2	98.8	84.98	97.88	90.01	99.27	87.93	98.33
Mild disabled	9.22	88.88	10.31	87.8	7.64	91.08	8.65	87.55
Severe disabled	6.17	91.67	9.02	86.14	4.05	91.1	6.92	86.29
CCI								
0	69.48	65.32	64.01	55.04	68.01	62.53	64.29	53.47
1	44.54	40.72	51.53	39.52	52.13	41.17	58.63	40.49
2	37.58	36.89	42.07	34.38	36.16	35.41	40.52	32.49
3~	43.49	51.99	58.47	51.2	45.58	50.93	58.4	49.17
Insurance Type								
The self-employed insured	31.41	71.72	34.55	70.3	16.64	70.82	18.21	66.63
The self-employed insured-dependent	11.67	61.91	16.18	64.87	24.87	65.88	26.15	64.87
The employed insured	38.56	77.18	29.36	70.42	24.59	68.12	21.7	64.61
The employed insured-dependent	42.2	77	45.55	77.53	54.72	80.36	55.98	80.67
Medical aid beneficiary	8.32	86.7	11.63	73.74	10.36	88.13	11.67	84.99
Medical aid beneficiary-dependent	1.08	72.44	1.05	61.8	2.67	73.91	2.68	66.96
	Overall SD	Between SD	Overall SD	Between SD	Overall SD	Between SD	Overall SD	Between SD
Household income	3.275	3.045	3.309	3.073	3.472	3.234	3.252	1.306

CCI = Charlson comorbidity index, SD = standard deviation.

Regarding changes in household income deciles in each group by gender with reference to time t (active TB diagnosis), household income markedly declined by about 0.143 (SE 0.039) after time t compared to before time t in men. In women, it slightly increased by 0.036 (SE 0.048), but the change was only within the margin of error (Table 3).

**Table 3:** Crude difference in differences of household income between the study groups by gender.

		Control group				TB group									
		Mean	Overall SD	Between SD	Within SD	Mean	Overall SD	Between SD	Within SD	Difference	SE	p	DID	SE	p
Men	Pre-treatment	5.690	3.270	3.194	0.697	5.683	3.269	3.194	0.698	−0.007	0.030	0.816			
	Post-treatment	5.744	3.278	3.122	1.056	5.594	3.337	3.188	1.109	−0.150	0.025	<0.001	−0.143	0.039	<0.001
Women	Pre-treatment	5.593	3.477	3.412	0.673	5.575	3.482	3.417	0.673	−0.018	0.037	0.632			
	Post-treatment	5.563	3.468	3.299	1.125	5.581	3.477	3.334	1.131	0.018	0.031	0.560	0.036	0.048	0.459

TB = tuberculosis, SD = standard deviation, SE = standard error, DID = difference in differences.

**Fig 2:**
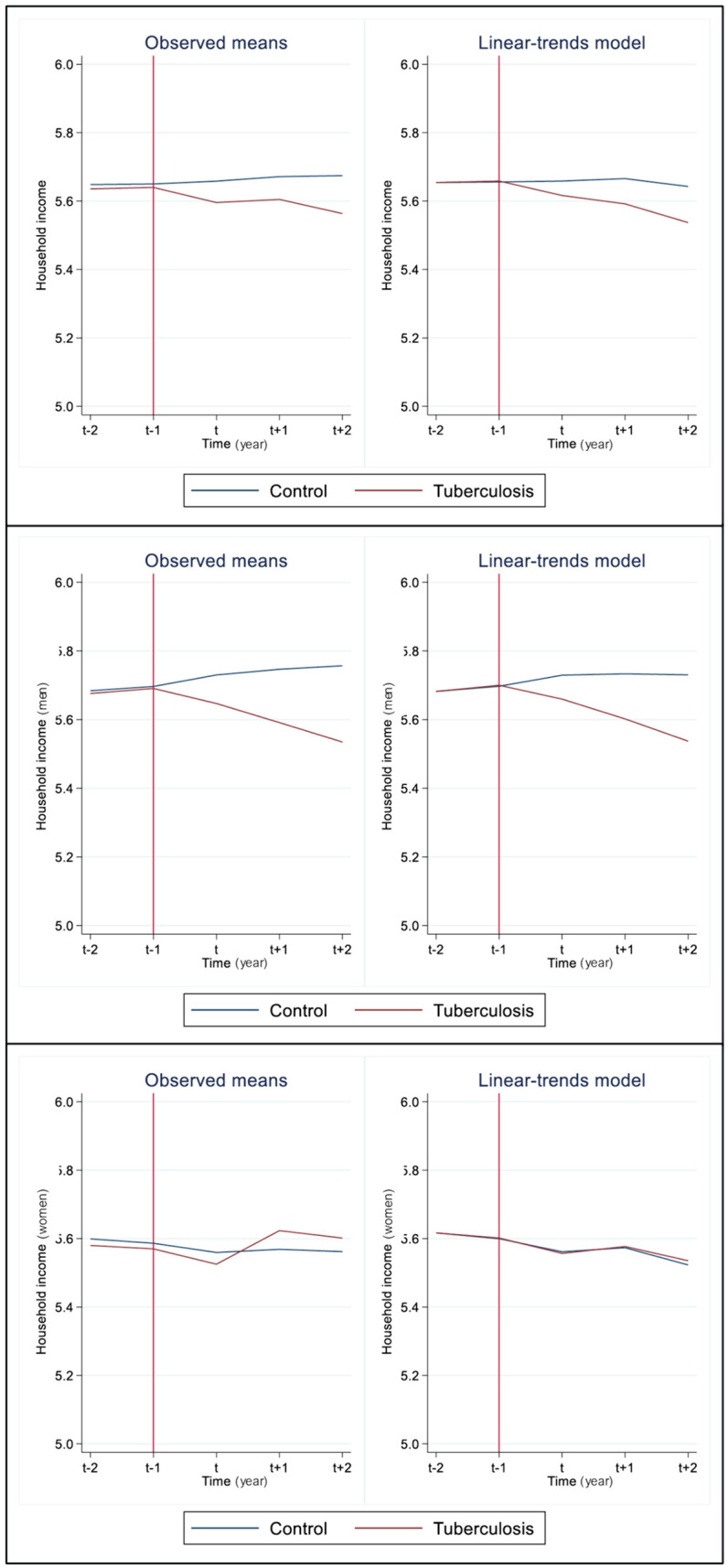
The trends of household incomes between the study groups by time. The left graphs are simple plots, and the right ones are linear-trend model. Vertical red line was the end of pre-treatment period.

**Fig 3:**
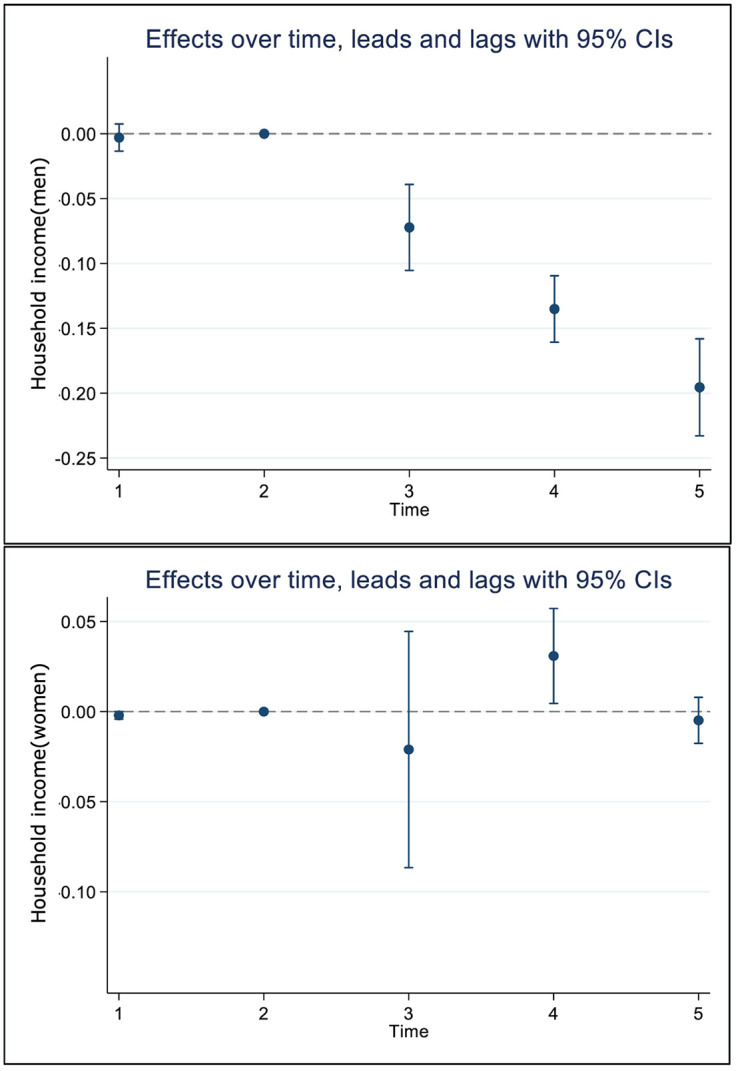
Time specific effects in the difference of household income by gender. The circles are coefficient, and the vertical lines are 95% confidence intervals. CI = confidence intervals.

Before examining treatment effects, we confirmed that the parallel trends assumption was satisfied. The treatment and control groups exhibited nearly identical trends in household income (Fig 2), and the event study coefficients at t–2 and t–1 were close to zero (Fig 3). In the panel DID model including covariates age, disability status, and CCI, household income decile decreased by about 0.131 (95% CI, model 1 = −0.146 ~ −0.115, model 2 = −0.132 ~ −0.129) post-treatment in the TB group compared to the control group in men, but there was no marked change in women (DID coefficient = 0.002, 95% CI, model 1 = −0.028 ~ 0.033, model 2 = −0.031 ~ 0.013) (Table 4, Fig 2). The analytical model satisfied the assumption of parallel trend (p-value = 0.175). In terms of the effects of treatment over time, household income gap between the control group and TB group was apparent from time t in men, and this trend continuously intensified until t + 2 (t + 2 DID coefficient = −0.196, 95% CI = −0.233 ~ −0.158). However, in women, the household income gap between the control group and TB group was not substantial, with the TB group actually having a higher income at t + 1 (t + 1 DID coefficient = 0.030, 95% CI = 0.005 ~ 0.014) (Table 5, Fig 3).

**Table 4:** Adjusted model of difference in differences between the study groups by gender.

		DID coefficient	95% CI	
Men	Model 1	−0.131	−0.146	−0.115
	Model 2		−0.132	−0.129
Female	Model 1	0.002	−0.028	0.033
	Model 2		−0.031	0.013

DID=difference in differences, CI=confidence intervals.

**Table 5:** Time-specific differences of household income between the study groups by gender.

	Men			Women		
	Coefficient	95% CI		Coefficient	95% CI	
Pre-treatment	−0.003	−0.013	0.008	−0.002	−0.004	0.000
t	−0.072	−0.105	−0.039	−0.021	−0.087	0.045
t + 1	−0.135	−0.161	−0.110	0.031	0.005	0.057
t + 2	−0.196	−0.233	−0.158	−0.005	−0.018	0.008

CI = confidence intervals.

**Fig 4:**
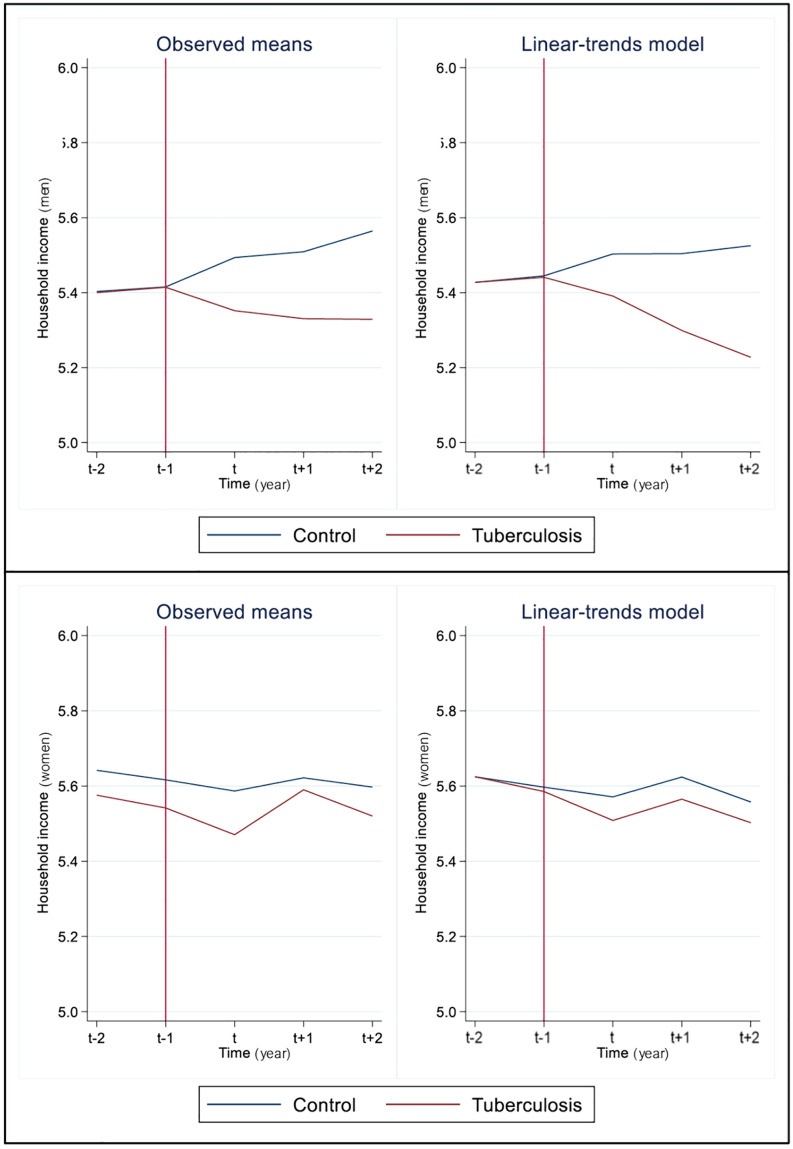
The trends of household incomes between the study groups by time among people who was 20 ~ 65 years old. The left graphs are simple plots, and the right ones are linear-trend model. Vertical red line was the end of pre-treatment period.

In the subgroup analysis of economically active populations (age 20–65 years) and only the employed insured, the results were different from those obtained in the primary analyses. Among individuals aged 20–65 years, household income in the TB group decreased by about 0.200 (95% CI = −0.202 ~ −0.199) compared to the control group in men. In a subgroup analysis of workers, household income in the TB group decreased compared to the control group in women (DID coefficient = −0.072, 95% CI = −0.110 ~ −0.034) (Table 6, Fig 4).

**Table 6:** Subgroup analysis of difference in differences by gender.

	Men				Women		
	Coefficient	95% CI		Coefficient		95% CI	
20 ~ 65 years old	−0.200	−0.202	−0.199	−0.053		−0.096	−0.010
The employed insured	−0.050	−0.066	−0.033	−0.072		−0.110	−0.034

CI = confidence intervals.

The results of three sensitivity analyses were generally in the same trend as those of the primary analyses. However, when the time of treatment was delayed by one year (t + 1), the household income in the TB group increased after treatment by about 0.021 (95% CI, model 1 = 0.021 ~ 0.022, model 2 = 0.021 ~ 0.021) compared to the control group in women (Table 7).

**Table 7:** Sensitivity analysis of difference in differences by gender.

		Men			Women		
		Coefficient	95% CI		Coefficient	95% CI	
Sensitivity 1	Model 1	−0.139	−0.156	−0.122	0.021	0.021	0.022
	Model 2		−0.282	−0.135		0.021	0.021
Sensitivity 2	Model 1	−0.070	−0.073	−0.067	0.027	−0.024	0.079
	Model 2		−0.070	−0.055		−0.027	0.045
Sensitivity 3	Model 1	−0.131	−0.146	−0.116	0.002	−0.026	0.030
	Model 2		−0.132	−0.129		−0.032	0.011

Sensitivity 1: treatment time was changed from t to t + 1; sensitivity 2: the lowest and highest household income group were excluded; sensitivity 3: drug-resistant tuberculosis cases was excluded. CI = confidence intervals.

## Discussion

Social mobility due to disease, which was our hypothesis, differed between women and men. For men, household income decile decreased by about 0.131 after TB diagnosis compared to control group, indicating approximately a 1.3 decile decline in income status. In contrast, there was no difference between the two groups for women. Differences in income changes between the two groups over time were also notable, with the gap in household income between the control and TB groups increasing over time for men, but no difference was found for women. Based on the subgroup analyses, however, it can be inferred that disease diagnosis in continuously working men and women follows the path of impoverishment. In women, shifts from being insured to the dependent on insurance eligibilities, influenced by changes in their work status after TB diagnosis, may decrease their household income.

Key finding of the study is that TB episode drives downward social mobility among both working women and men although those patters differ each other. The trend of household income reduction due to disease was continuously strengthened for three years from the time of disease diagnosis in working men. Household income slightly increased after one year of disease diagnosis but again dropped two years after disease diagnosis in working women. These results commonly suggest that income decline due to active TB cannot be overcome in a short period of time and that it may worsen in the long run. Study findings pertaining to trends of downward social mobility due to an illness are not consistent. Previous study in Korea that followed the trajectory of impoverishment due to a health shock reported that the odds for impoverishment increases until two years after the year of disease diagnosis and declines from three years after diagnosis [7]. Previous studies conducted in European countries among working-age population have reported consistent findings, showing that the odds of job loss and retirement increase after disease diagnosis in both men and women [28,29]. On the other hand, a study in United States investigating social mobility from a health shock observed no marked trend of income decline in individuals with prolonged disease duration [30]. Unfortunately, there were not many longitudinal studies on patients with active TB, a chronic infectious disease and poverty-causing disease, but Choi and his colleagues [22] examined inter-generational social mobility in patients with TB observed that the odds for lower household income were high at time of TB and continued to be higher over several years in the group with low parental SES. Therefore, it is possible that the downward mobility due to active TB may follow a chronic trajectory.

Those long-run influence of disease on downward social mobility urgently calls for universal social protection as TB strategy. Korea has gaps in social protection measures for TB patients, particularly in addressing income security during and after TB treatment. Current TB related social protection predominantly focuses on direct medical cost coverage while comprehensive social protection needs such as income support remain inadequately addressed. Although living allowances are available for some TB patients, the strict means-testing eligibility criteria result in very few patients actually receiving these benefits. Importantly, the Korean society lacks paid sick leaves and sickness cash benefits, which hinders reducing the degree of social suffering due to an illness [8]. Such structural limitations also serve as a cause of increased catastrophic total costs due to TB in many low- and middle-income countries [14]. Therefore, health and social protection policies may be important strategies for preventing social distress due to an illness, such as TB. Based on this, social protective strategies have been adopted as a core tuberculosis strategy employed by the WHO [31]. The WHO targets to attain 0% in terms of households suffering from catastrophic total costs due to TB as part of its End TB Strategy, and social protection strategy is a major priority [13]. According to a recent systematic review, about 43% (95% CI 34–51) of households face catastrophic total costs due to TB, but one limitation of this data is that most included studies were conducted in low- to middle-income countries [14].

Our findings revealed gender differences in the effects of TB episode on social mobility. The implications of TB episode for income reduction, especially among employed women, raise significant questions about women’s disadvantaged and underprivileged economic position in and out their family in Korea. The primary breadwinner within a household in Korea is still socially defined as the male spouse, with the female spouse playing a secondary role in livelihood [32]. Our data show that among the male study population, 49.9% are insurers and 42.2% are dependents, whereas among women, the figures are 26.3% insurers and 64.2% dependents. Women are more disadvantaged in the Korean labor market compared to men, as exemplified by the largest gender pay gap among OECD countries [33]. Their jobs also consist of more precarious employment relationships than their counterparts [34,35]. Women’s multifaceted employment precariousness renders them disproportionately vulnerable to the entitlement for social protection [34]. Thus, when women are sick, they are more likely to be pressured to leave labour market and driven to be a dependent of their other family members, an employed spouse or employed children. In addition, household income, which has raised after a TB diagnosis among women in our findings, may also partially support it. The association between changes in position within the labor market and path of impoverishment due to disease episode could be estimated based on the distributions in the panel data as well. For instance, when the employed-insured women diagnosed TB, about 17% of them changed their category of insurance changed the employed or self-employed insured-dependents while only 9% of control group changed.

This study has a few limitations. First, household income was measured as income deciles classified based on national health insurance contribution levels and individuals’ status defined for health insurance contribution. That is, if two or members of a single household have an income above a certain threshold, they are considered to be separate households due to their primary work-based health insurance subscriber status. Thus, this should be taken into account when interpreting the meaning of household income in the present study. In addition, we treated income deciles as a continuous outcome in our regression models, which may not fully satisfy equal-interval assumptions. While this approach is defensible for ordinal variables with many categories and approximately normal distributions [36], future research should employ ordered regression models to confirm our findings. Secondly, there was no parameter that directly measures employment status in our study data. Although wage earners could be identified based on their health insurance eligibility status, we could not include labor with low income and informal labor. Because of these limitations, we could not measure mobility within the labor market as a major mechanism of social mobility and instead conducted subgroup analyses of working-age population and wage earners in the formal labor domain. Third, we could not include partnership status, which may affect gender gaps in social mobility, in our analysis. Importantly, these limitations may obscure the dynamic fluctuations in household members’ economic status when a patient is diagnosed with TB and potentially underestimate gender inequalities in these dynamics. If employment status and partnership status information had been available, we could have explored more specific mechanisms underlying our findings. For instance, the lack of significant income declines effects among women following TB episodes might be attributed to the “added worker effect [37],” whereby the health condition of one household member influences household income by prompting adjustments in the economic activities of other members, particularly spouses [38–40]. This mechanism could explain why the economic impact of TB appears differently across genders in our analysis. Therefore, our findings may underestimate the true economic burden of TB on women, as household-level income measures might mask individual-level income losses when other household members compensate through increased labor participation. Future studies should incorporate individual-level employment data, household composition information, and qualitative interviews to better capture the mechanisms underlying gender differences in TB’s economic impact. Given that sufficient data sources for such comprehensive analyses are not currently available, prospective cohort studies with longer follow-up periods are needed to provide more comprehensive understanding of how TB affects household economic dynamics across genders.

## Conclusion

This study showed that there is a marked trend of declining income due to the diagnosis and treatment of active TB in men but not in women. This discrepancy may be attributable to the differences in gender roles in a patriarchal society and higher possibility of women moving out of the labor market after disease. The results showing a trend of declining income in women in subgroup analysis of only the working-age population and employees support this possibility. There is a pressing need for more gender-responsive and universal implementation of health and social protection policies in order to alleviate the trend of social suffering caused by disease.
